# A Frontier Review of Semantic SLAM Technologies Applied to the Open World

**DOI:** 10.3390/s25164994

**Published:** 2025-08-12

**Authors:** Le Miao, Wen Liu, Zhongliang Deng

**Affiliations:** School of Electronic Engineering, Beijing University of Posts and Telecommunications, Beijing 100876, China; miaole@bupt.edu.cn (L.M.); dengzhl@bupt.edu.cn (Z.D.)

**Keywords:** semantic SLAM, open-world perception, multimodal sensor fusion, zero-shot learning, robotic perception

## Abstract

With the growing demand for autonomous robotic operations in complex and unstructured environments, traditional semantic SLAM systems—which rely on closed-set semantic vocabularies—are increasingly limited in their ability to robustly perceive and understand diverse and dynamic scenes. This paper focuses on the paradigm shift toward open-world semantic scene understanding in SLAM and provides a comprehensive review of the technological evolution from closed-world assumptions to open-world frameworks. We survey the current state of research in open-world semantic SLAM, highlighting key challenges and frontiers. In particular, we conduct an in-depth analysis of three critical areas: zero-shot open-vocabulary understanding, dynamic semantic expansion, and multimodal semantic fusion. These capabilities are examined for their crucial roles in unknown class identification, incremental semantic updates, and multisensor perceptual integration. Our main contribution is presenting the first systematic algorithmic benchmarking and performance comparison of representative open-world semantic SLAM systems, revealing the potential of these core techniques to enhance semantic understanding in complex environments. Finally, we propose several promising directions for future research, including lightweight model deployment, real-time performance optimization, and collaborative multimodal perception, and offering a systematic reference and methodological guidance for continued advancements in this emerging field.

## 1. Introduction

With the continuous advancement of modern technology, robotics has gradually become an integral part of our daily lives [1]. To enable robots to perform tasks autonomously, one of the primary challenges is to solve the problem of robot self-localization and navigation [2]. Simultaneous localization and mapping (SLAM) technology provides robots with the ability to autonomously perceive and act in unknown environments by solving the joint optimization problem of real-time pose estimation and environmental map construction based on online observations and data association [3]. The introduction of semantic information further bridges the gap from geometric understanding to cognitive reasoning [4,5,6].

Semantic SLAM relies on predefined, closed semantic label libraries (e.g., “chair”, “door”) and uses supervised learning to achieve object recognition and map annotation. However, the closed-world assumption faces fundamental challenges in dynamic and complex open-world environments: on the one hand, the limited number of predefined semantic categories fails to cover the long-tail distribution of objects encountered in the real world [7,8]—such as ad hoc rescue equipment in disaster zones or emerging smart appliances in households; on the other hand, static label libraries are unable to adapt to the dynamic nature of evolving semantic concepts. These limitations severely restrict the practical applicability and generalization capabilities of semantic SLAM in fields such as service robotics and augmented reality. To address the challenges of limited categories and insufficient semantic generalization in traditional semantic SLAM systems, open-world semantic SLAM has emerged as a research hotspot in recent years [9,10,11,12]. This direction aims to enhance the robustness of SLAM systems in dynamic and complex scenes that contain unknown categories, enabling the system not only to perform simultaneous localization and mapping but also to perceive and understand novel semantic information.

Open-world semantic SLAM is a simultaneous localization and mapping technology designed for open environments, aimed at overcoming the dependency on predefined semantic labels, allowing the system to recognize novel categories and adapt to unknown environmental changes. In recent years, researchers have increasingly focused on improving key performance aspects of semantic SLAM systems in open-world scenarios, such as localization accuracy, semantic generalization, and real-time capabilities. To address these challenges, studies have widely integrated techniques such as zero-shot learning [13,14,15,16], dynamic semantic expansion [17,18,19,20,21,22], and multimodal semantic fusion [17,23,24], thereby enhancing the system’s ability to understand semantics and construct maps in complex, dynamic environments. Based on these advancements, this paper provides a detailed review and performance comparison of representative open-world semantic SLAM approaches across the three core dimensions mentioned above.

The main contributions of this paper are as follows:

- Section 2 organizes and introduces the development and current research status of open-world semantic SLAM technology, summarizing the progress and significance of semantic SLAM in open-world settings based on existing studies;
- Section 3 focuses on the three key technologies of open-world semantic SLAM: zero-shot open-vocabulary understanding, dynamic semantic expansion, and multimodal semantic fusion. It systematically reviews the cutting-edge advancements and core challenges, aiming to provide theoretical guidance and technical roadmaps for future research and to promote the transition of semantic SLAM from controlled lab environments to real-world open environments;
- Section 4 compiles commonly used datasets and evaluation metrics for open-world semantic SLAM, and evaluates and compares the performance of current algorithms in related studies;
- Section 5 presents future research directions and recommendations.

## 2. The Evolution from Visual SLAM to Open-World Semantic SLAM

**Figure 1:**
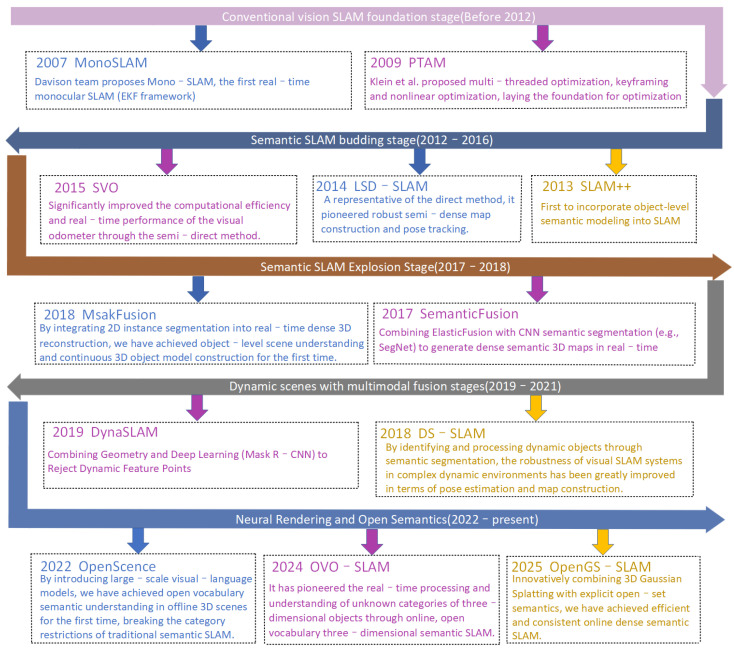
Technological evolution of open-world semantic SLAM.

As illustrated in Figure 1, the evolution of SLAM clearly reflects the paradigm shift in robotic systems from purely geometric perception to deep environmental understanding. Early SLAM systems, such as MonoSLAM [25] and PTAM [26], primarily focused on constructing sparse or dense geometric maps in unknown environments, with the core goal of solving the robot’s self-localization and environmental structure reconstruction problems. Building on this foundation, researchers further explored methods to improve mapping density and efficiency. For instance, LSD-SLAM [27], as the first semi-dense direct SLAM system capable of operating in large-scale environments, relied on image gradient information rather than explicit feature extraction, significantly enhancing mapping density. Simultaneously, SVO [28] combined sparse feature tracking with image gradient information, achieving a balance between high precision and real-time performance, and became a foundational module for many subsequent systems. However, despite breakthroughs in geometric reconstruction density and efficiency, the maps produced by these systems were still primarily constituted of spatial geometric information, lacking a high-level cognitive understanding of object categories, attributes, and functions within the environment. This limitation severely restricted the robots’ ability to perform complex reasoning and human–robot interaction.

To address this gap, researchers began to integrate semantic information into the SLAM framework. Pioneering works such as SLAM++ [29] explored the possibility of combining object-level semantic priors with map construction. Subsequently, systems such as MaskFusion [30] and SemanticFusion [31] enhanced semantic understanding and map interpretability by utilizing pretrained models for semantic segmentation or mapping the outputs of convolutional neural networks to 3D dense maps. However, these early semantic SLAM methods faced a common inherent limitation: they heavily relied on predefined, closed-set category labels. This means that the system could only recognize and track a limited number of categories that were known during the training phase. When encountering new objects in real-world open environments that were not learned during training, the system’s generalization capability would sharply decline, leading to the so-called “semantic rigidity” problem. This significantly hindered the widespread application of semantic SLAM in complex real-world scenarios. To tackle the challenges posed by dynamic objects, systems such as DS-SLAM [32] and DynaSLAM [33] introduced motion consistency checks and multi-view geometry constraints to eliminate dynamic regions. Although this improved localization robustness to some extent, their reliance on supervised semantic segmentation and the assumption of low scene dynamics still limited their adaptability when faced with highly dynamic and complex real-world environments.

In the 2020s, with the rise of large-scale vision-language pretraining models such as CLIP (contrastive language-image pretraining) [34], the research on semantic SLAM underwent a paradigm shift, officially advancing into the open-world domain. Research during this phase aimed to overcome the limitations of closed-set categories and enable systems to achieve zero-shot open-vocabulary understanding. These large models successfully aligned visual features with natural language descriptions of open vocabulary through contrastive learning on large-scale image–text pairs, granting SLAM systems unprecedented zero-shot recognition capabilities. As a result, systems could now understand and recognize new categories that had never appeared in the training data. This breakthrough led to a series of pioneering works, such as OpenScene [35] and CLIP-Fields [36]. OpenScene embedded CLIP features into 3D point clouds for the first time, achieving zero-shot recognition of open vocabulary and greatly expanding the system’s category generalization capabilities. Meanwhile, CLIP-Fields explored the incremental expansion of dynamic semantic knowledge bases via weak supervision mechanisms, allowing the system to continuously learn and update its semantic knowledge over time, effectively addressing dynamic interference and new concept adaptation during long-term scene evolution. Despite these significant advancements, methods in the open-world semantic SLAM phase still face the inherent trade-off between openness and accuracy. Specifically, the CLIP model is susceptible to contextual ambiguity and visual-semantic similarity interference in complex 3D scenes, leading to category recognition confusion (e.g., misclassifying a “circular charger” as a “teacup”). Additionally, the high computational overhead of large models limits the system’s real-time performance. Moreover, the incremental update mechanism for the semantic knowledge base is still in its infancy, and weakly supervised models lack stability in handling frequent category switching and semantic conflicts. To overcome these challenges, subsequent research continues to focus on advancements. OVO-SLAM [37], the first system to support online open-vocabulary semantic mapping, integrates CLIP features with SLAM processes to enable real-time responses to arbitrary semantics. Building on this, OpenGS-SLAM [38] further introduced 3D Gaussian Splatting, creating a unified technical architecture that integrates efficient semantic rendering, mapping, and label consistency optimization under open set conditions.

To more intuitively illustrate the differences in technical characteristics and evolutionary directions between visual SLAM and open-world semantic SLAM methods, particularly their developments in sensor applications and semantic processing approaches, Table 1 presents a comparison of the key methods in terms of sensor types and semantic mechanisms used throughout the evolution from traditional visual SLAM to open-world semantic SLAM. Looking back at the evolution of semantic SLAM, it is clear that open-world semantic SLAM, compared to traditional frameworks, has achieved a qualitative leap from “recognizing the known” to “understanding the unknown” by introducing online incremental learning mechanisms, zero-shot open vocabulary understanding, and multimodal (especially vision-language) collaborative perception. This paradigm shift not only solves the dynamic interference and semantic rigidity problems encountered during long-term scene evolution but also endows systems with the ability to continuously adapt to environmental changes, dynamically expand semantic knowledge, and support natural language interactions. This has laid a solid technological foundation for the seamless integration of robots into the complex and ever-changing real world.

## 3. Theoretical Foundations and Key Enabling Technologies

In open-world environments, robots are confronted with increasingly complex challenges, primarily characterized by dynamic changes, the frequent emergence of previously unseen semantic entities, and the pressing need for the integration of multi-source heterogeneous information [39]. These challenges significantly elevate the demands placed on semantic SLAM systems in terms of perception, understanding, and reasoning capabilities [40]. Traditional semantic SLAM approaches—due to their inherent closed-set recognition paradigm, static semantic modeling frameworks, and unimodal perception mechanisms—are becoming inadequate for effectively addressing the diversity, temporal evolution, and multimodal complementarity of semantics in open-world scenarios. To overcome the above technical bottlenecks, this chapter will focus on three key technologies: zero-shot open-vocabulary understanding, dynamic semantic expansion, and multimodal semantic fusion. Specifically, zero-shot open-vocabulary understanding leverages cross-modal semantic alignment models to enable generalized inference over novel semantic categories, fundamentally eliminating reliance on predefined closed label sets [41]. Dynamic semantic expansion incorporates temporal awareness and incremental modeling techniques, empowering semantic maps with the capability to continuously adapt and update in dynamic environments [42]. Meanwhile, multimodal semantic fusion aims to synergistically integrate diverse modalities—such as vision, language, and geometry—through cross-modal collaborative optimization, thereby substantially enhancing the system’s perceptual robustness and semantic comprehension in complex and uncertain environments. These three core technologies are highly interdependent and collectively establish the theoretical foundation and technical framework for enabling robust and efficient perception and understanding in open-world semantic SLAM. This chapter systematically analyzes their fundamental principles, recent research advances, and intrinsic interconnections, laying solid groundwork for subsequent discussions on practical applications and future research directions.

### 3.1. Zero-Shot Open-Vocabulary Understanding

Zero-shot open-vocabulary understanding has emerged as a critical perceptual paradigm for addressing the challenges posed by unknown semantic entities in open-world environments. At its core lies the principle of cross-modal semantic alignment [43,44]. Leveraging large-scale pretrained vision-language models (VLMs), this approach maps visual features into a shared semantic embedding space, thereby overcoming the reliance on predefined category labels inherent in conventional closed-set recognition methods [45]. The fundamental mechanism involves contrastive learning on massive image–text pairs, enabling VLMs to implicitly capture and learn deep semantic associations between visual and textual modalities. As a result, semantically related entities from different modalities—such as an image of a “teacup” and the textual phrase “a cup”—are projected close to each other in the joint embedding space [46]. This unified representation empowers systems to perform semantic inference and recognition of previously unseen categories directly from natural language descriptions, without the need for additional fine-tuning. Consequently, the semantic generalization capability extends from limited training sets to open-ended domains, allowing the seamless integration of novel semantic concepts into geometric maps, and enabling the construction of interpretable and hierarchically structured semantic topologies.

**Figure 2:**
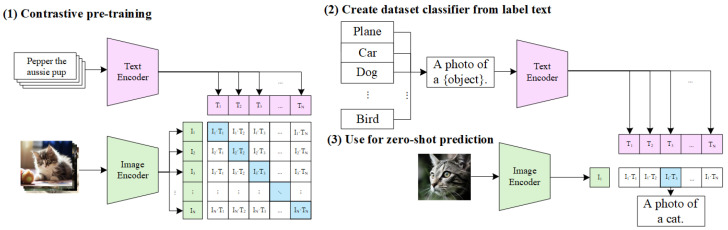
CLIP model diagram.

Recently, the emergence of the CLIP model has provided a powerful technical foundation for realizing zero-shot open-vocabulary understanding. CLIP employs large-scale contrastive learning on image–text pairs to align visual and linguistic features in a unified high-dimensional embedding space, significantly advancing open-vocabulary semantic understanding tasks [47]. As illustrated in Figure 2, CLIP consists of an image encoder—typically based on ResNet [48] or Vision Transformer (ViT) [49] and a text encoder based on the Transformer architecture [50]. These two encoders operate collaboratively, transforming input images and natural language descriptions into high-dimensional feature vectors. By optimizing a contrastive loss function that minimizes the embedding distance for matched image–text pairs while maximizing it for mismatched pairs, CLIP effectively learns to capture fine-grained cross-modal semantic relationships. This mechanism enables open-world semantic SLAM systems to perform zero-shot classification and semantic recognition directly guided by natural language prompts, significantly enhancing adaptability and generalization in complex, dynamic, and unpredictable real-world environments. From a fundamental principle perspective, the learning mechanism of the CLIP model can be viewed as a symbolic-level alignment reasoning process: images and text serve as symbolic carriers in different modalities, respectively. After being encoded by their respective encoders and embedded into a shared semantic space, they undergo semantic pairing optimization through contrastive loss. This approach is not merely a shallow feature mapping but a higher-order cross-modal knowledge alignment method, exhibiting certain ‘symbolic reasoning’ characteristics—that is, the model does not merely memorize the superficial forms of images or text but abstracts generalizable semantic concepts and expresses them discriminatively in a unified space. Furthermore, the contrastive embedding space constructed by CLIP is essentially a distribution-based semantic knowledge graph, enabling inference and matching through inner product or similarity metrics, analogous to a formalized logical reasoning mechanism. This process shares similarities with propositional matching in computational logic and word sense disambiguation in computational linguistics, indicating that CLIP not only achieves breakthroughs at the engineering level but also possesses cognitive rationality, providing theoretical support for language-driven perception in embodied intelligent systems.

Meanwhile, more vision-language pretrained frameworks, such as BLIP-2 [45] and LLaVA [51], have demonstrated exceptional cross-modal reasoning capabilities. BLIP-2 leverages a lightweight image encoder in conjunction with a large language model for collaborative learning, significantly enhancing zero-shot generation and understanding abilities, making it suitable for deployment on resource-constrained platforms. LLaVA, on the other hand, combines visual perception with multi-turn dialogue capabilities, enabling open-ended understanding and question answering of complex image content, thereby providing SLAM systems with the potential for language-driven semantic interpretation.

Significant efforts have been made to extend zero-shot capabilities to 3D scene understanding. A seminal work in this area is OpenScene [35], which pioneered the transfer of CLIP’s pretrained visual-linguistic knowledge to 3D point clouds, enabling the zero-shot recognition of arbitrary open-vocabulary categories. Specifically, OpenScene associates each 3D point with a CLIP-derived feature vector, allowing objects in the 3D scene to be queried and labeled using free-form natural language descriptions. This facilitates the segmentation and understanding of previously unseen semantic concepts in 3D environments directly via textual prompts. To further enhance the applicability of zero-shot open-vocabulary understanding in practical SLAM systems, recent research has increasingly focused on real-time, online, and dynamically adaptive methods. A notable advancement in this direction is OVO-SLAM [37], the first online open-vocabulary semantic SLAM system. OVO-SLAM achieves the deep integration of CLIP features within the SLAM mapping pipeline, enabling real-time responses to arbitrary semantic queries. Its core mechanism involves the online detection and tracking of 3D semantic segments while computing and aggregating corresponding CLIP embeddings. This approach significantly broadens the system’s category generalization capability and outperforms several offline methods in both segmentation accuracy and runtime efficiency. Building on this foundation, OpenGS-SLAM [38] introduces a novel integration of 3D Gaussian Splatting (3DGS) as a scene representation, resulting in a unified framework that combines efficient semantic rendering, mapping, and label consistency optimization under open-set conditions. By explicitly embedding open-vocabulary semantic labels—derived from 2D vision-language models—into the 3D Gaussian representation, OpenGS-SLAM achieves notable improvements in semantic rendering speed and memory efficiency.

Despite these advances, the practical deployment of zero-shot open-vocabulary understanding in open-world semantic SLAM systems remains challenged by several critical issues. A fundamental limitation lies in the trade-off between openness and recognition accuracy. Vision-language models such as CLIP may suffer from semantic ambiguities in complex 3D scenes due to contextual noise [52], varying viewpoints, and visual-semantic similarity among categories. Additionally, the substantial computational overhead intrinsic to large models continues to pose a major barrier to achieving truly real-time, efficient systems [53]. Future research is expected to focus on developing more lightweight and robust semantic embedding techniques, exploring strategies for effective aggregation of multi-view or multi-frame semantic information, and incorporating scene-level contextual reasoning to enhance both the zero-shot open-vocabulary understanding and the overall robustness of systems operating in dynamic and uncertain environments.

### 3.2. Dynamic Semantic Expansion

Dynamic semantic expansion is a critical enabler for open-world semantic SLAM systems, designed to empower them with the ability to adaptively update and extend their semantic understanding over time and across varying environments [54,55]. In open-world settings, the semantics of scenes and objects are inherently non-stationary—new object instances may emerge, object positions may shift, and semantic categories may evolve dynamically. Such variability poses significant challenges for traditional SLAM systems, which often assume a fixed set of known classes and static semantic priors [56]. To address these challenges, dynamic semantic expansion is closely integrated with zero-shot open-vocabulary understanding. By constructing a continuously updated semantic knowledge base and performing the real-time re-identification of objects in the environment, SLAM systems can incrementally refine and broaden their semantic comprehension during long-term operation. This capability not only demands the robust recognition and interpretation of static objects but also emphasizes the efficient real-time processing, reasoning, and incorporation of novel semantic concepts within a dynamic 3D environment. The ultimate goal is to continuously evolve the system’s knowledge representation, enabling it to accommodate new semantic concepts and maintain interpretability and coherence within the map.

Within the realm of open-world semantic SLAM, dynamic semantic expansion has emerged as a foundational technique to empower systems with the capacity to recognize, interpret, and integrate previously unseen semantic entities not present in the training set. This significantly enhances their generalization ability and adaptability in complex, dynamic, and unstructured environments. Recent advances have primarily focused on embedding open-vocabulary recognition into real-time SLAM systems. For instance, OVO-SLAM [37] represents a major milestone by integrating CLIP features directly into the SLAM mapping pipeline, enabling the real-time zero-shot recognition of unknown 3D semantic segments. This method has significant advantages in terms of real-time performance and generalization capabilities, but it has certain limitations in incremental learning and semantic drift control. OVO-SLAM adopts a frame-by-frame inference strategy, which lacks long-term semantic knowledge accumulation and is prone to catastrophic forgetting when new objects continuously appear. OpenGS-SLAM [38] innovatively uses 3D Gaussian splatting to dynamically map 2D labels to 3D space and achieves the efficient semantic mapping and updating through Gaussian voting splatting technology. This method effectively reduces label drift problems encountered in open environments by combining 2D label consensus and semantic knowledge adaptive updating. However, OpenGS-SLAM still faces the challenge of maintaining semantic consistency and stability when encountering large-scale environmental changes, especially under the conditions of significant perspective changes or severe occlusions. PanoSLAM [57] enhances semantic stability during prolonged operation by introducing a spatio-temporal consistency module. Through this module, PanoSLAM not only stably transfers 2D semantic labels to 3D scenes but also effectively suppresses label drift caused by dynamic environments. Nevertheless, PanoSLAM’s incremental learning capabilities have not been fully demonstrated, and how to effectively accumulate new knowledge during long-term operation while preventing semantic drift and catastrophic forgetting remains a challenge. SNI-SLAM [58] enhances incremental learning capabilities by introducing neural implicit representations and cross-attention mechanisms, enabling the gradual updating and fusion of new and old semantic information in dynamic environments. The advantage of SNI-SLAM lies in its adaptive feature fusion method, which can flexibly handle semantic updates in different environments. Additionally, it reinforces the continuous learning and optimization of semantics through feature loss strategies, making it highly robust when facing complex scenes.

Despite these advances, dynamic semantic expansion in practical open-world SLAM applications still faces several core challenges. The foremost issue lies in achieving robust recognition and long-term consistency for previously unseen semantic categories. Systems must accurately identify and stably annotate novel concepts in highly dynamic and ambiguous environments, while avoiding semantic drift over time [59]. In addition, achieving the efficient and adaptive online incremental learning of semantics—without compromising performance or causing catastrophic forgetting—remains an open problem. The system must also be capable of semantic disambiguation under conditions of label ambiguity, ensuring semantic robustness and coherence as the environment evolves.

### 3.3. Multimodal Semantic Fusion

With the rapid advancement of foundational vision-language models (e.g., CLIP), LiDAR-based semantic perception, event cameras, and other heterogeneous sensing technologies, open-world semantic SLAM systems are evolving from single-modality perception to collaborative multimodal sensing [60,61]. In complex, dynamic environments where unknown objects and categories frequently emerge, synergistic reasoning and mutual compensation across multiple modalities have become essential for mitigating partial perception failures and enhancing environmental adaptability [62].

**Figure 3:**
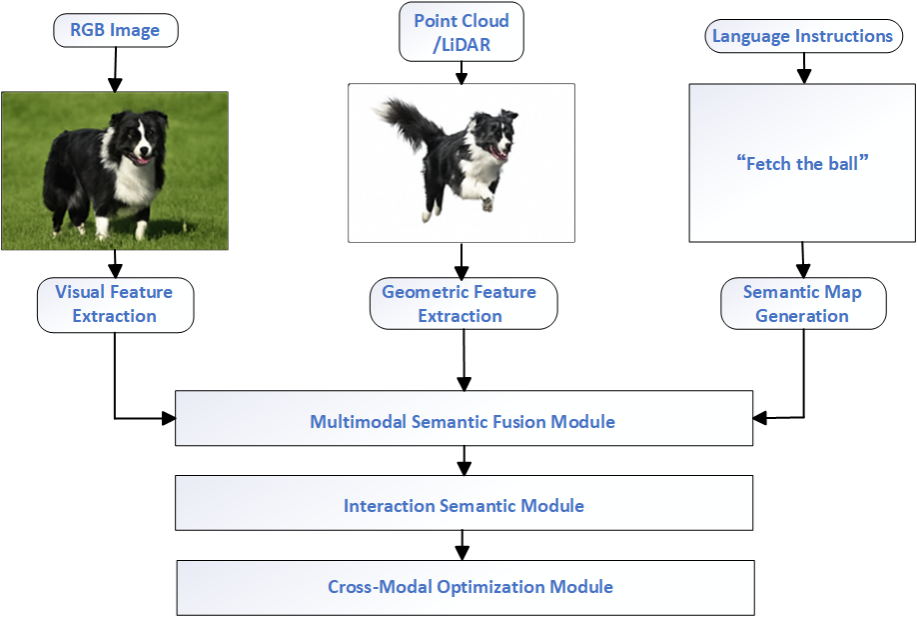
Multimodal interaction mechanism.

Against this backdrop, multimodal semantic fusion has emerged as a pivotal technique in open-world semantic SLAM, aiming to harmonize the representation and reasoning of visual, linguistic, and spatial-geometric information [63]. Diverse sensors—including RGB-D cameras, LiDAR, IMUs, event cameras, and speech/language interfaces—play a foundational role in acquiring complementary information, thus enriching the multimodal feature fusion pipeline. As illustrated in Figure 3, a typical multimodal interaction framework encompasses synchronized perception across sensors, cross-modal feature alignment and reasoning, and the construction of semantically enhanced maps. This architecture highlights how multimodal inputs collaboratively compensate for perception degradation caused by occlusions, motion blur, or semantic ambiguity in real-world conditions. By integrating synergistic mechanisms across modalities—such as vision-language, image-point cloud, and text-space interactions—the system not only alleviates limitations in isolated modalities but also facilitates more robust semantic mapping and task planning. Importantly, this fusion framework extends beyond synchronized sensor input, enabling dynamic integration and optimization of multimodal observations in a unified semantic embedding space through semantically enriched map representations.

In recent years, the field of open-world semantic SLAM has made significant progress in multimodal fusion and interaction optimization, profoundly changing the traditional SLAM system’s assumptions about static environments and reliance on single-modality input. The core of current research lies in how to efficiently integrate information from different sensors and modalities, and achieve complementary advantages through sophisticated interaction mechanisms, so as to cope with the challenges of complex, dynamic, and unstructured environments. The main advances are reflected in three aspects: First, the efficient fusion of multi-source heterogeneous data is the foundation. By explicitly integrating open-vocabulary semantic labels from 2D vision models into 3D geometric representations [38], the cross-modal transfer of semantic knowledge and the semantic enhancement of 3D scenes have been achieved. Second, the deep interaction and optimization between semantics and geometry is the core for improving system robustness. For example, OVO-SLAM [37] deeply integrates CLIP features with the SLAM mapping process, enabling the system to respond to arbitrary semantic queries and perform zero-shot segmentation in real time. This interactive optimization forms a closed-loop mechanism in which semantics and geometry mutually promote each other. Finally, the intelligence and adaptability of fusion mechanisms have become a development trend. SNI-SLAM [58] uses neural implicit representation and cross-attention mechanisms to achieve the adaptive fusion of appearance, geometry, and semantic features, as well as hierarchical semantic understanding. These advances together lay a solid foundation for building more intelligent and robust autonomous systems.

These frontier studies have jointly promoted the continuous development of multimodal semantic fusion technology in the field of open-world semantic SLAM, making it more robust and adaptive in coping with unknown environments, dynamic changes, and real-time requirements, and laying a solid foundation for the development of key application fields such as intelligent robotics and autonomous driving [64]. However, the practical application of multimodal semantic fusion in open-world semantic SLAM still faces many challenges. First, the heterogeneity of data and the difficulty of alignment are core problems [65,66]. The data from different modal sensors have significant differences in sampling frequency, spatial resolution, noise characteristics, and data representation. How to achieve accurate temporal synchronization, spatial alignment, and effective information complementation is still a complex problem [67]. Especially in dynamic environments, the asynchrony between sensors and motion blur will further increase the difficulty of alignment.

## 4. Algorithm Evaluation and Performance Comparison

### 4.1. Open-World Dataset

In the development of semantic SLAM systems for open-world scenarios, the selection of datasets plays a decisive role in evaluating algorithm performance and generalization capabilities. Traditional SLAM datasets are typically constructed with closed-set category definitions, which limits their ability to reflect the diverse and dynamic semantic distributions found in real-world environments. As a result, a new generation of datasets with open-world characteristics has emerged in recent years. As shown in Table 2, this work analyzes and compares several representative datasets, including OpenScene [35], OpenVocab-3D, SemanticKITTI, ScanNet, and Replica. In the “Openness Level” column, a high rating denotes support for unknown category recognition, open-vocabulary labeling, and zero-shot learning; medium indicates fixed categories with extensibility, instance-level annotations, or 3D support; low refers to datasets limited to closed-world semantic tasks.

OpenScene is a multimodal dataset designed for open-world semantic understanding, supporting the fusion of RGB, depth maps, and natural language descriptions. It aims to address the incompleteness of predefined category sets. By incorporating open-vocabulary annotations, OpenScene significantly enhances a SLAM system’s ability to recognize unseen categories. Similarly, OpenVocab-3D introduces large-scale vision-language models such as CLIP into the 3D perception domain [68], embedding natural language labels into point clouds. It establishes a benchmark platform with zero-shot semantic generalization capabilities, making it one of the most critical datasets in current open-vocabulary SLAM research.

In contrast, classical datasets such as SemanticKITTI and ScanNet are built upon fixed-category taxonomies. SemanticKITTI, derived from the KITTI LiDAR sequences, provides point-level semantic labels. Although it follows a closed-set category design, its large scale and complex urban scenarios make it a widely adopted benchmark for dynamic semantic mapping. On the other hand, ScanNet, an indoor RGB-D dataset, offers rich scene structures and dense semantic annotations. It is well-suited for studying semantic reconstruction and localization in static environments, though it exhibits limitations in terms of openness and category diversity.

Replica deserves special mention as it comprises high-fidelity reconstructed scenes from real-world scans, featuring dense semantic and instance-level annotations. It has been extensively utilized in various semantic SLAM systems, especially those leveraging Gaussian splatting and neural rendering techniques, such as OVO SLAM, SNI SLAM, and Pano SLAM. Although Replica’s semantic labels are still based on a fixed category set, its realism, controllability, and support for cross-modal mapping make it a valuable supplement for evaluating open-vocabulary semantic SLAM systems.

In summary, these datasets offer distinct advantages in terms of modality variety, semantic openness, and annotation granularity, collectively providing multidimensional support for advancing open-world semantic SLAM research. In future developments, establishing unified benchmarking standards and enabling cross-task transfer using these datasets will be key directions for further investigation.

### 4.2. Performance Metrics

In the field of open-world semantic SLAM, the comprehensive and accurate evaluation of algorithms is essential for driving technological advancement [69,70]. To quantitatively assess and compare the performance of different methods, researchers typically focus on a set of key evaluation metrics [71]. This section highlights three core indicators: Absolute Trajectory Error (ATE), Mean Intersection over Union (mIoU), and Frames Per Second (FPS). These metrics, respectively, reflect localization accuracy, semantic understanding capability, and real-time performance, offering a multidimensional basis for evaluating the effectiveness and robustness of open-world semantic SLAM algorithms.

#### 4.2.1. Absolute Trajectory Error

In open-world semantic SLAM systems, evaluating localization accuracy is a critical component of performance analysis. ATE is a widely adopted metric for assessing the global consistency between the estimated and ground truth trajectories [72]. ATE is computed by temporally aligning the estimated and ground truth poses based on their timestamps, followed by calculating the root mean square error (RMSE) across the entire trajectory. The result is often visualized through trajectory plots, offering a clear and quantitative evaluation of the SLAM system’s localization performance over time [73].

Specifically, let the estimated camera poses obtained by the system be $P_{1},\ldots ,P_{n}\in SE\left(3\right)$, and the corresponding ground truth poses be $Q_{1},\ldots ,Q_{n}\in SE\left(3\right)$, where each represents a similarity transformation from the estimated pose to the ground truth pose. The ATE for the i-th frame is then defined as

(1) $$F_{i}:=Q_{i}^{-1}SP_{i}$$

To compute the overall performance, we use the root mean square error (RMSE), which aggregates the errors over all frames:

(2) $$\mathrm{RMSE}\left(F_{1:n},\Delta \right):={\left(\frac{1}{m}\sum_{i=1}^{m}{∥\mathrm{trans}(F_{i})∥}^{2}\right)}^{\frac{1}{2}}$$

#### 4.2.2. Mean Intersection over Union

mIoU is a widely used evaluation metric in semantic segmentation tasks, designed to assess the pixel-level accuracy of class predictions. In open-world semantic SLAM systems, mIoU is employed to evaluate the consistency between the generated semantic maps and the actual scene, thereby reflecting the system’s capability to understand and reconstruct semantic information within the environment [74].

For each semantic class *c*, the intersection over union (IoU) is defined as the ratio of the intersection to the union between the predicted region and the ground truth:

(3) $${\mathrm{IoU}}_{c}=\frac{|{\mathrm{Prediction}}_{c}\cap {\mathrm{GroundTruth}}_{c}|}{|{\mathrm{Prediction}}_{c}\cup {\mathrm{GroundTruth}}_{c}|}$$

The mean intersection over union (mIoU) is calculated as the average IoU across all semantic classes:

(4) $$\mathrm{mIoU}=\frac{1}{C}\sum_{c=1}^{C}{\mathrm{IoU}}_{c}$$

where *C* denotes the total number of semantic classes. The value of mIoU ranges from 0 to 1, with higher values indicating better segmentation performance.

#### 4.2.3. Frames per Second

In open-world semantic SLAM, frames per second (FPS) serves as a direct indicator of a system’s real-time performance and computational efficiency [75]. FPS measures the number of frames the system can process or generate per unit time, and it reflects the model’s runtime throughput on a given hardware platform. The FPS is computed as

(5) $$\mathrm{FPS}=\frac{N_{\mathrm{frames}}}{T_{\mathrm{total}}}$$

where $N_{\mathrm{frames}}$ denotes the total number of processed frames and $T_{\mathrm{total}}$ represents the total elapsed processing time in seconds.

In intelligent systems such as autonomous vehicles and service robots, which operate in highly dynamic and unpredictable environments, FPS is as critical as ATE and mIoU. It directly reflects the system’s ability to perform real-time perception, decision making, and execution under specific hardware constraints, thus playing a pivotal role in practical deployment scenarios.

### 4.3. Performance Comparison

In recent years, high-performance open-world semantic SLAM systems have made substantial progress across multiple dimensions, particularly in terms of localization accuracy, semantic generalization, and real-time efficiency. This section presents a comparative analysis of several representative systems from these key perspectives, highlighting their respective strengths and limitations. Such a comparison aims to facilitate a deeper understanding of the current state of the art, emerging trends, and the practical applicability of semantic SLAM technologies in diverse real-world scenarios. To ensure the comprehensiveness and scientific rigor of the reviewed literature, this study conducted literature searches based on multiple international authoritative academic databases and publishing platforms, including but not limited to Web of Science, IEEE Xplore, and the ICRA conference proceedings. During the search process, several core keywords were defined based on the research theme, such as ‘semantic SLAM,’ ‘open world,’ ‘sensors,’ ‘zero-shot,’ and ‘multimodal,’ with the time scope limited to 2020 and beyond. All retrieved literature underwent preliminary screening, abstract evaluation, and full-text review. Based on ensuring quality and relevance, only literature with high academic contributions and innovation in research methods, experimental design, and conclusions was ultimately included for performance comparison.

#### 4.3.1. Accuracy Improvement

From the perspective of accuracy improvement, SNI-SLAM [58] introduces a multimodal feature fusion framework combined with hierarchical semantic representations. By leveraging a cross-attention mechanism,

(6) $$T_{s}=softmax\left(\frac{f_{g}f_{a}^{T}}{\sqrt{\|f_{a}{\|}_{2}^{2}}}\right)f_{s}$$

(7) $$T_{a}=softmax\left(\frac{f_{g}\cdot T_{s}^{T}}{\sqrt{\|T_{s}{\|}_{2}^{2}}}\right)f_{a}$$

the system jointly integrates geometric, semantic, and appearance features maintains tracking robustness even when lighting changes or segmentation fails, enabling collaborative optimization. This approach significantly enhances the performance of these three feature types during the mapping process, resulting in more detailed and consistent semantic segmentation outcomes.

In this framework, geometric features $f_{g}$, appearance features $f_{a}$, and semantic features $f_{s}$ are jointly modeled. The semantic representation $T_{s}$ is adaptively weighted based on the matching consistency between geometric and appearance features, resulting in a fused semantic representation. Simultaneously, the appearance features are refined via a correlation mechanism with both the geometric and fused semantic features, yielding enhanced appearance features $T_{a}$.

Moreover, SNI-SLAM employs a coarse-to-fine dual-layer feature plane, where the coarse-grained layer captures the global semantic layout and the fine-grained layer encodes local structural details, enabling scalable mapping across large-scale environments. Experiments on the Replica dataset demonstrate a tracking error as low as 0.456 cm, underscoring the system’s high accuracy.

Similarly, OpenGS-SLAM [38] introduces an explicit Gaussian point representation combined with a confidence-aware fusion strategy to further enhance object-level mapping precision, particularly in densely structured scenes. The proposed Confidence-based 2D Label Consensus mechanism effectively resolves multi-view label inconsistency under open-set conditions. Additionally, segmentation-aware pruning based on object instance counts improves semantic boundary accuracy.

(8) $${\bar{C}}_{s}=\frac{\sum_{l_{s}^{i}\in P_{s}^{j}}C_{s}^{i}\times |l_{s}^{i}|}{\sum_{l_{s}^{i}\in P_{s}^{j}}|l_{s}^{i}|}$$

Specifically, partial match confidence is computed by evaluating the average confidence over the area of the partially matched label set $P_{s}^{j}$. Experimental results demonstrate that the proposed method achieves a tracking error of 0.16 cm on the Replica dataset.

PanoSLAM [57] is the first to incorporate panoptic segmentation into a 3D Gaussian splatting-based SLAM system, pioneering a unified approach to geometric, semantic, and instance-level reconstruction in open-world environments. To address the challenge of pseudo-label noise under open-set conditions, it further integrates a spatio-temporal label enhancement (STL) module. This framework is designed to enable robust and comprehensive 3D panoptic scene reconstruction in complex, unconstrained settings. Its key contributions are two-fold:

1. Extended 3D Gaussian representation: The conventional Gaussian Splatting framework is extended to a 13-dimensional parameter space, enabling the joint optimization of geometric, semantic, and instance-level attributes. This enriched representation supports multimodal rendering, including depth, color, semantics, and instance labels;
2. Spatio-temporal label enhancement (STL) module: To address the noise inherent in pseudo-labels generated by pretrained 2D vision models, the STL module projects these labels into a 3D voxel space. By leveraging multi-view consistency, it performs joint optimization using a voxel-wise label aggregation formulation
  (9) $$\hat{R}\left(P^{*}\right)=\frac{1}{|g_{n}|}\sum_{*\in g_{n}}\hat{R}\left(P^{*}\right)$$
  and a unified loss function,
  (10) $$L=\sum_{t,P}\left[\lambda_{1}L_{1}\left(C_{t},C_{GT}\right)+\lambda_{2}L_{1}\left(D_{t},D_{GT}\right)+\lambda_{3}CE\left(O_{t},{\hat{O}}_{t}\right)+\lambda_{4}DICE\left(R_{t},{\hat{R}}_{t}\right)\right]$$
  thereby enabling annotation-free, panoptic 3D reconstruction under open-world conditions.

This method eliminates the need for manual annotations by leveraging the open-vocabulary capabilities of pretrained 2D vision models, thereby significantly reducing dependency on labeled datasets. However, its accuracy is still affected by fluctuations in semantic consistency. On the Replica dataset, it achieves an mIoU of 50.67% and a tracking error of 0.39 cm.

In contrast, OVO-SLAM [25] emphasizes open-vocabulary semantic augmentation by dynamically updating semantic labels through CLIP-guided embeddings. While its accuracy is slightly lower than that of SNI-SLAM and OpenGS-SLAM, it demonstrates superior generalization capabilities in terms of category scalability and open-set recognition.

#### 4.3.2. Semantic Generalization Ability

**Figure 4:**
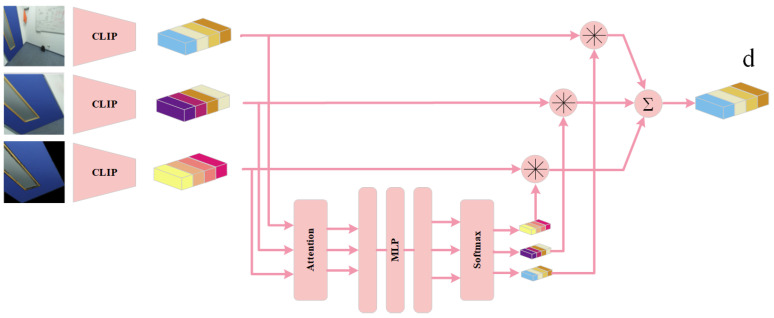
CLIP merger.

In terms of semantic generalization, OVO-SLAM overcomes the vocabulary constraints inherent in traditional semantic SLAM systems. As the first online, real-time 3D semantic SLAM framework with open-vocabulary capabilities, it has attracted significant attention. The system can recognize arbitrary object categories without relying on a predefined vocabulary and operates independently of ground-truth pose or geometric priors, substantially expanding the applicability of semantic SLAM in real-world scenarios. At the core of OVO-SLAM is its dynamic CLIP descriptor fusion module (CLIP Merger). As illustrated in Figure 4, for each 2D mask, three types of CLIP-based feature vectors are extracted: a global image descriptor, a background-free mask descriptor, and a bounding-box descriptor including background context. These vectors are then adaptively weighted and fused via a self-attention network to generate the final descriptor *d*. This fusion mechanism enables the system to select the most representative descriptor for each 3D instance from the optimal viewpoint, facilitating robust multi-view open-vocabulary semantic aggregation.

OVO-SLAM demonstrates exceptional 3D semantic segmentation performance on standard datasets such as Replica and ScanNet, achieving state-of-the-art results. Notably, this method represents the first online semantic SLAM system operating in open-vocabulary scenes without relying on real pose or geometric ground truth information, thus significantly expanding the system’s perceptual and application boundaries in unknown environments.

In contrast, OpenGS-SLAM combines 2D semantic models with Gaussian point clouds to support the explicit modeling of unknown object classes, excelling in open-set recognition. While PanoSLAM does not incorporate open vocabulary, its unsupervised adaptation capability to unlabeled data remains highly significant for real-world scenarios. SNI-SLAM, although offering rich semantic representations, still relies on a closed-set label source, slightly limiting its generalization capability.

#### 4.3.3. Real-Time Performance Optimization

Finally, in terms of real-time performance optimization, both NeRF-Based SLAM [76] and OVO-SLAM demonstrate superior system responsiveness. Building upon the NICE-SLAM framework [77], NeRF-Based SLAM introduces depth $d_{n}$ uncertainty modeling for RGB-D measurements, which enables local precision enhancement through weighted error minimization. Specifically, it updates the depth loss calculation formulas in the mapping and tracking threads based on *N* pixels per grid resolution as follows:

(11) $$L_{\mathrm{depth}}^{\mathrm{map}}=\frac{1}{N}\sum_{n=1}^{N}\frac{{∥d_{n}-{\hat{d}}_{n}∥}_{1}}{\sigma_{n}^{d}}$$

(12) $$L_{\mathrm{depth}}^{\mathrm{track}}=\frac{1}{N}\sum_{n=1}^{N}\frac{{∥d_{n}-{\hat{d}}_{n}∥}_{1}}{\sqrt{\sigma_{n}^{d^{2}}+{\hat{\sigma }}_{n}^{d^{2}}}}$$

Here, $\sigma_{n}^{d}$ is the standard deviation of $d_{n}$, and ${\hat{\sigma }}_{n}^{d}$ corresponds to the standard deviation of ${\hat{d}}_{n}$, which is the depth measurement at pixel *n*. For simplicity, assuming that $d_{n}$ and ${\hat{d}}_{n}$ are independent, the uncertainties of these two variables are then combined.

Moreover, the NeRF-Based SLAM system leverages motion priors from the IMU using the following formulation.

(13) $$L_{\mathrm{track}\;\mathrm{IMU}}=e^{C_{\mathrm{IMU}}^{T}}\Sigma_{C_{\mathrm{IMU}}}^{-1}e^{C_{\mathrm{IMU}}}+e^{r_{\mathrm{IMU}}^{T}}\Sigma_{r_{\mathrm{IMU}}}^{-1}e^{r_{\mathrm{IMU}}}$$

to optimize camera poses and employs the pre-integrated relative motion increment (RMI) in the mapping thread.

(14) $$L_{\mathrm{map}\;\mathrm{RMI}}=\sum_{q=2}^{\mathrm{len}(z)}e^{{\mathrm{RMI}}^{T}}z\left[q-1\right]z\left[q\right]\Sigma_{{\mathrm{RMI}}^{-1}}z\left[q-1\right]z\left[q\right]e^{\mathrm{RMI}}z\left[q-1\right]z\left[q\right]$$

It enhances pose consistency between keyframes. Experiments show that the integration of IMU significantly improves the convergence speed under resource-constrained conditions. The system improves the SLAM framework’s adaptability in unknown and dynamic environments while maintaining computational efficiency.

In contrast, OVO-SLAM maintains high efficiency in simultaneous mapping and semantic processing through a lightweight semantic update mechanism. While both SNI-SLAM and OpenGS-SLAM achieve superior mapping accuracy, their adoption of complex cross-modal fusion architectures or Gaussian point projection mechanisms incurs substantial computational overhead, limiting their applicability on resource-constrained platforms. PanoSLAM represents an intermediate solution, relying on vision foundation models for semantic inference; however, its inference performance is constrained by the scale and parallelism of the underlying model.

In summary, Table 3 lists the performance comparison of these classic open-world semantic SLAM methods. Since some methods did not report their complete performance metrics in the original paper, the missing parts are indicated by ‘-’ in the table. SNI-SLAM and OpenGS-SLAM excel in semantic accuracy and fine-grained reconstruction, making them suitable for applications demanding high-fidelity mapping. In contrast, OVO-SLAM and NeRF-based SLAM demonstrate advantages in real-time performance and adaptability, better aligning with deployment requirements in mobile or open environments. PanoSLAM, by offering an unsupervised panoramic mapping paradigm, achieves a practical balance between semantic capability and system efficiency in real-world applications.

## 5. Summary and Prospects

Semantic SLAM enhances traditional SLAM systems by incorporating high-level semantic information, significantly enriching map representations and environmental understanding. It has become a pivotal research direction for advancing autonomous localization and scene perception. In open-world scenarios, where environments are typically dynamic, unstructured, and subject to long-term changes, SLAM systems face elevated demands in adaptability, robustness, and real-time performance. Open-world semantic SLAM addresses these challenges by integrating techniques such as zero-shot open-vocabulary understanding, dynamic semantic expansion, and multimodal semantic fusion. These approaches compensate for the limitations of purely geometric representations, improving localization accuracy, semantic generalization, and real-time capabilities in complex environments.

Despite promising progress, several critical challenges remain. First, zero-shot recognition in complex 3D environments entails a trade-off between openness and precision. Current vision-language models often struggle with category ambiguity due to contextual confusion, viewpoint variation, and semantic similarity, compromising perception reliability. Moreover, their large-scale architectures pose difficulties for real-time inference on resource-constrained platforms. Second, dynamic semantic expansion requires the robust recognition and consistent mapping of previously unseen semantic categories. SLAM systems must support incremental identification and the continual learning of novel semantics while mitigating catastrophic forgetting and semantic drift to ensure long-term operational stability. Third, multimodal fusion in open-world scenarios remains hindered by data heterogeneity and alignment challenges. Disparities in sensor sampling rates, resolutions, and noise profiles complicate temporal synchronization, spatial registration, and feature-level fusion, particularly in dynamic environments—representing key technical bottlenecks.

To address these issues, future research should focus on

- Designing robust semantic representation mechanisms tailored for open-vocabulary scenarios, incorporating multi-view, multi-frame, and contextual modeling strategies to mitigate inter-class confusion and semantic ambiguity;
- Developing efficient and lightweight vision-language models and edge deployment frameworks that balance recognition accuracy with computational efficiency;
- Constructing semantic expansion frameworks that support continual and incremental learning, incorporating memory-based architectures and regularization techniques to prevent catastrophic forgetting and enhance adaptability to novel categories;
- Improving multimodal fusion by advancing temporal-spatial alignment and cross-modal feature matching methods, alongside uncertainty modeling and robust state estimation, to enhance system stability and perception performance in dynamic open-world settings.

Research on open-world semantic SLAM is not only of theoretical significance but also shows broad prospects in a variety of practical application scenarios. For example, service robots need to identify newly appearing objects and dynamic layouts in home or commercial environments; in space exploration missions, robots must perform autonomous mapping and scene understanding in completely unknown environments; and in intelligent warehousing and logistics, robots must cope with the dynamic nature of the environment and semantic changes caused by frequent changes in personnel and items. These application requirements further drive the development of open-world semantic SLAM systems toward higher adaptability, generalization capabilities, and autonomy.

In summary, open-world semantic SLAM will continue to make breakthroughs in open vocabulary understanding, dynamic semantic expansion, and multimodal fusion, providing more reliable environmental perception and adaptive capabilities for key application scenarios such as intelligent robots, autonomous driving, and augmented reality, and driving the development of next-generation intelligent systems with long-term autonomy and semantic understanding capabilities.

## Figures and Tables

**Table 1:** A structured comparison of key methods from traditional visual SLAM to open-world semantic SLAM.

Method	Monocular	RGB-D	No Semantic	Closed-Set Semantic	Open-World Semantic
MonoSLAM	✓	×	✓	×	×
PTAM	✓	×	✓	×	×
LSD-SLAM	✓	×	✓	×	×
SVO	✓	×	✓	×	×
SLAM++	×	✓	×	✓	×
SemanticFusion	×	✓	×	✓	×
DS-SLAM	×	✓	×	✓	×
DynaSLAM	×	✓	×	✓	×
OVO-SLAM	×	✓	×	×	✓
OpenGS-SLAM	×	✓	×	×	✓

**Table 2:** Commonly used datasets in open-world semantic SLAM.

Dataset	RGB	LiDAR	Depth	Language	Instance Labels	Indoor	Outdoor	Openness Level
OpenScene	✓	×	✓	✓	✓	✓	✓	High
OpenVocab-3D	✓	×	×	✓	✓	✓	×	High
SemanticKITTI	×	✓	×	×	×	×	✓	Medium
ScanNet	✓	×	✓	×	✓	✓	×	Medium
Replica	✓	×	✓	×	✓	✓	×	Medium

**Table 3:** Performance comparison of classic algorithms in open-world semantic SLAM.

Method	Contribution	ATE	mIoU (%)	FPS	System	Code Resource
NeRF-Based SLAM [76]	Extending the NeRF baseline approach to open-world scenarios	-	-	-	NVIDIA RTX 2070 SUPER GPU	-
SNI-SLAM [58]	Proposing multimodal feature fusion and hierarchical semantic coding	0.456	87.41	2.15	NVIDIA RTX 4090 GPU	https://github.com/IRMVLab/SNI-SLAM (accessed on 10 August 2025)
OVO-SLAM [37]	First online open vocabulary 3D semantic SLAM implementation	-	27.1	-	NVIDIA RTX 3090 GPU	-
PanoSLAM [57]	First implementation of geometric reconstruction, 3D semantic segmentation and instance segmentation in a unified framework	0.39	50.67 (Avg)	-	NVIDIA RTX 4090 GPU	https://github.com/runnachen/PanoSLAM (accessed on 10 August 2025)
OpenGS-SLAM [38]	Implementing dense semantic SLAM in open set scenarios	0.16	63.17	165.47	-	https://young-bit.github.io/opengs-github.github.io/ (accessed on 10 August 2025)

## Data Availability

Not applicable.
